# Opisthorchiasis: An Overlooked Danger

**DOI:** 10.1371/journal.pntd.0003563

**Published:** 2015-04-02

**Authors:** Ludmila M. Ogorodova, Olga S. Fedorova, Banchob Sripa, Viatcheslav A. Mordvinov, Aleksei V. Katokhin, Jennifer Keiser, Peter Odermatt, Paul J. Brindley, Oleg A. Mayboroda, Thirumalaisamy P. Velavan, Maxim B. Freidin, Alexey E. Sazonov, Irina V. Saltykova, Mariya Y Pakharukova, Yulia V. Kovshirina, Kostas Kaloulis, Olga Y. Krylova, Maria Yazdanbakhsh, the TOPIC Consortium

**Affiliations:** 1 Siberian State Medical University, Tomsk, Russian Federation; 2 Tropical Disease Research Laboratory, Department of Pathology, Faculty of Medicine, Khon Kaen University, Khon Kaen, Thailand; 3 Laboratory of Molecular Mechanisms of Pathological Processes, Institute of Cytology and Genetics, Siberian Branch of the Russian Academy of Sciences, Novosibirsk, Russian Federation; 4 Department of Medical Parasitology and Infection Biology, Swiss Tropical and Public Health Institute, Basel, Switzerland; 5 University of Basel, Basel, Switzerland; 6 Department of Epidemiology and Public Health, Swiss Tropical and Public Health Institute, Basel, Switzerland; 7 Department of Microbiology, Immunology and Tropical Medicine, and Research Center for Neglected Diseases of Poverty, School of Medicine & Health Sciences, George Washington University, Washington, D.C., United States of America; 8 Center for Proteomics and Metabolomics, Leiden University Medical Center, Leiden, the Netherlands; 9 Department of Chemistry, Tomsk State University, Tomsk, Russian Federation; 10 Institute of Tropical Medicine, University of Tübingen, Tübingen, Germany; 11 Academic Division of Thoracic Surgery, Royal Brompton Hospital, London, United Kingdom; 12 Population Genetics Laboratory, Research Institute for Medical Genetics, Siberian Branch of Russian Academy of Medical Sciences, Tomsk, Russian Federation; 13 Department of Natural Sciences, Novosibirsk State University, Novosibirsk, Russian Federation; 14 ReMedys Foundation, Geneva, Switzerland; 15 External R&D Innovation, Pfizer Russia, Moscow, Russian Federation; 16 Department of Parasitology and Leiden Parasite Immunology Group, Leiden University Medical Center, Leiden, the Netherlands; University of Queensland, AUSTRALIA

## Background

A group of helminth infections, caused by liver flukes of the trematode family Opisthorchiidae, were recently the focus of discussions at a meeting where scientists from Russia, Southeast Asia, Europe, and the United States came together in Tomsk city in Western Siberia (Russia) to form a Tomsk OPIsthorchiasis Consortium (TOPIC). This initiative starts a platform to raise awareness, to strengthen integrated control, and to conduct research on a neglected infectious disease that afflicts populations not only in the tropical regions of East Asia but also in temperate and semi-arctic areas of Europe and Asia [1].

The Opisthorchiidae of importance to humans are *Opisthorchis felineus*, *Opisthorchis viverrini*, and *Clonorchis sinensis*, each of which has a discrete, though occasionally overlapping, geographical distribution: *O*. *felineus* is endemic in Europe and Russia; *C*. *sinensis* in China, the Republic of Korea, and northern Vietnam; and *O*. *viverrini* in Southeast Asia. Together they affect more than 45 million people worldwide [2]. Human infection with *O*. *felineus* results from eating raw or undercooked freshwater fish carrying the metacercariae of the parasite. The ingested larvae develop further and migrate to the bile ducts by chemotaxis, where adult worms feed on biliary epithelia and contents in the bile. Adult worms shed eggs that enter the gastrointestinal tract and are released with the faeces to the external environment. Freshwater snails of the family Bithyniidae ingest the eggs and, following the release of the miracidia from the eggs, several stages of development take place within the snail until cercariae have developed. Shed cercariae can penetrate freshwater fish, where they encyst in the skin or flesh [3].

Within the genus *Opisthorchis*, *O*. *felineus* is the species with the highest zoonotic potential, which has important implications not only for veterinary medicine but also for maintenance of transmission to humans even under high hygienic standards in which the risk of freshwater contamination by human faeces is low [4]. The morbidities associated with opisthorchiasis are largely hepatobiliary, specifically stemming from bile duct fibrosis and cholangitis, and are expressed in a variety of manifestations, such as obstructive jaundice, hepatomegaly, abdominal pain, and nausea [5]. Importantly, there are studies in animal models supported by other epidemiologic data that indicate that *O*. *viverrini* and *C*. *sinensis* infections can lead to cholangiocarcinoma, a generally incurable and, hence, fatal bile duct cancer [6,7], which has resulted in the classification of these parasites to the Group 1 carcinogens by the International Agency for Research on Cancer [1,8,9]. Despite the unarguable public health importance of these infections, both in terms of numbers of humans infected worldwide and clinical impact, it has been given relatively little recognition by health authorities, grant-giving agencies, and the pharmaceutical industry. There has been an incremental increase in awareness following seminal work in Thailand, Korea, and, later, in China, Laos, and Cambodia, where a number of studies have clarified the situation regarding epidemiology, pathogenesis, and control. Notably, genome sequence information on these liver flukes is increasingly available, for example, at www.trematode.net [10]. However, important gaps remain and, in particular, there is a paucity of information regarding *O*. *felineus*.

Therefore, an initiative was taken to organize a meeting in Tomsk, a city located in the region of Western Siberia that is highly endemic for *O*. *felineus* [4,11], and bring together a multidisciplinary cadre of investigators working on the Opisthorchiidae and infections caused by these fish-borne liver flukes. This event was welcomed by Pfizer, a multinational pharmaceutical company that supported the meeting, and that recognized the importance of infection with *O*. *felineus* as a neglected health threat in Russia. The meeting aimed to highlight the ongoing public health activities and research on diseases caused by Opisthorchiidae and, in particular, to identify the gaps in our knowledge of the epidemiology, clinical profile, treatment, and fundamental mechanisms of host–parasite interaction.

Scientists presenting and discussing their research findings covered a spectrum of topics that included epidemiology and clinical aspects of these infections, as well as aspects of host–parasite interaction and the molecular biology of these parasites.

## Epidemiology, Clinical Features, and Treatment

The body of data available on the epidemiology of Opisthorchiidae comes largely from studies conducted on infections with *O*. *viverrini*, and increasingly with *C*. *sinensis*, in which robust data have been, and are being, collected in Southeast Asia to map the endemic regions and to quantify the extent of the morbidity associated with the infections [2]. Peter Odermatt from the Swiss Tropical and Public Health Institute discussed the importance of this infection in rural areas of Thailand, Laos, Cambodia, and Vietnam [12]. In particular, the infection is widespread in Laos, where more than half of the population is infected; in highly endemic villages, 70% of the population is infected. Based on microscopy, 50% of the population in some villages in Laos was found to be infected with *O*. *viverrini*, with an increasing prevalence of infection with age [13]. Close correlation has been found between consumption of fish and prevalence of *O*. *viverrini* in communities in rural Laos where 23 different species of fish in the region can be infected, and, in some villages, up to 60% of some of these fish species carry the metacercariae [14]. Examination of cats and dogs that are often coinhabitants of households revealed that 30% of these domestic animals were infected with *O*. *viverrini*, and, thus, can contribute to intensifying transmission of these parasites to humans. Furthermore, the deeply culturally rooted habit of raw fish dish consumption is a major public health challenge [15].

Data collected on the clinical symptoms have provided a comprehensive view on the morbidities associated with *O*. *viverrini*, reporting that increasing intensity of infection and multiparasitic infection was associated with increasing reported symptoms of abdominal discomfort and disease in endemic communities [16–18]. The use of ultrasonography in the field has enabled conduct of large-scale examination of inhabitants of affected villages to accurately delineate the abnormalities associated with the infection [19]. In the infected population examined so far, normal hepatobiliary images generally were not observed, whereas mild and severe pathology with bile duct dilation was recorded in a high proportion of the adult population in rural areas [20].

The association between *O*. *viverrini* and cholangiocarcinoma, or bile duct cancer, was discussed by Banchob Sripa from Khon Kaen University, Thailand. Not only is the highest incidence of this bile duct cancer in the world seen in Northeast Thailand, where *O*. *viverrini* is highly endemic, but there is also compelling evidence from models using experimental infection of laboratory rodents demonstrating the close association between the liver fluke and cancer [21]. Whereas there is also considerable evidence for the association of *C*. *sinensis* and bile duct cancer, so far it is unknown whether *O*. *felineus* shares this feature, a clear area in which further research is warranted [1].

There are ongoing efforts to understand the risk factors for the development of severe morbidity including the bile duct cancer: coinfection with other parasitic or viral (hepatic) infections or smoking may be important. Large multi-centre and multi-country studies are needed to accurately map parasitic infection and risk factors for morbidity, including biomarkers for cholangiocarcinoma. Remote sensing and spatial Bayesian statistics that allow mapping of infection based on geophysical characteristics of the endemic areas are important tools to bring sociodemographic and environmental factors into the picture and, hence, prepare large-scale correlation maps of various risk factors and morbidity, including bile duct cancer [13]. At the biological level, metabolic phenotyping of subjects at risk of developing cholangiocarcinoma using body fluids was explained by Oleg Mayboroda from Leiden University Medical Center, the Netherlands. The use of enabling analytical technologies such as mass spectrometry (MS) and Nuclear Magnetic Resonance spectroscopy (NMR), now more accessible than before, can be helpful for early detection as well as understanding the mechanisms that lead to the development of cancer in infected subjects. Another area for application of the technologies is identification of metabolic profiles that are specific for Opisthorchiidae, as already done for other parasitic infections [22–24], which might then allow the development of simple field applicable rapid tests.

Currently, the treatment of Opisthorchiidae is based on giving praziquantel at 40 mg/kg body weight. Data presented by Peter Odermatt indicated that it might be necessary to increase the dose of praziquantel to 75 mg/kg, but at the risk of increasing adverse events, which is an important factor for community compliance [25]. The search for new therapies that are more effective and have reduced adverse events has come up with potential new drugs, such as tribendimidine [26–28].

## Host–Parasite Interaction

A number of basic mechanisms of host–parasite interaction regarding pathology and persistence were discussed. Banchob Sripa presented data on the ability of a number of parasite-derived molecules to drive uncontrolled growth of host cells and, thus, explain the possible association between *O*. *viverrini* and bile duct cancer. Molecules such as parasite-derived granulin, which leads to proliferation of biliary and other mammalian cells [29], or parasite-derived enzymes thioredoxin (TRX) and thioredoxin peroxidase (TPX), which prevent apoptosis, could be involved in stimulating uncontrolled growth of host cells [3]. Moreover, there are data indicating that *O*. *viverrini* extracts are able to stimulate inflammatory cytokines, such as IL-6 and IL-8 by human cholangiocytes, peripheral blood mononuclear cells, and that a higher level of IL-6 is seen in infected patients with bile duct cancer compared to those without [30–32]. Together, these data raise the question of whether the other Opisthorchiidae have strong proinflammatory properties that would explain the increased risk of cancer upon infection with these trematodes and open possibilities for prevention.

Linked to the inflammatory response induced by *O*. *viverrini*, Paul Brindley from George Washington University, Washington, D.C., US, discussed the alteration in the gut and bile microbiome of infected hosts. He presented findings that reveal that infection with *O*. *viverrini* modified the gut microbiome in hamsters. This investigation detected the unexpected presence of communities of very select species of bacteria in the bile of infected hamsters. Intriguingly, a number of species of environmental bacteria, and therefore not expected in the gut microflora, seemed to find their way into the bile along with *O*. *viverrini*. Might these microbes play an important role in driving chronic inflammation in the bile tract? These bacterial species were dissimilar to endosymbiotic *Neorickettsia* species known to be associated with trematodes at large [33]. Nonetheless, given that laboratory contamination can impact sequence-based microbiome analyses, especially in analysis of biofluids discrete from gut contents and faeces and where microbiota might be sparse [34], these findings will need to be confirmed in other settings. If, indeed, *O*. *viverrini* metacercariae and/or developing adult flukes vector environmental or exotic microbes into the biliary tree, persistent inflammatory responses to this fluke-associated microbiota could be a key factor in the development of cancer [3,19,21,35]. This report has paved the way for human studies to test whether similar mechanisms could be at play.

The basic immunological profile in humans infected with these parasites was the subject of the presentations by Olga Fedorova from Siberian State Medical University and Maria Yazdanbakhsh from Leiden University Medical Center, the Netherlands. In comparison with other parasitic helminths, relatively little has been done on the immunological profiling of humans infected with Opisthorchiidae, especially beyond *O*. *viverrini* infections [30]. The immunoepidemiological studies in the region of Tomsk comparing subjects infected with and without *O*. *felineus* has indicated that there is TH2 skewing as evident from elevated IgE in infected subjects compared to uninfected, but that the levels are much lower than what is found in studies of helminth infected subjects in Ghana where schistosomiasis is highly prevalent or in Indonesia in communities with geohelminth infections. This could result from the fact that intensity of infection is lower in *O*. *felineus* infected subjects and that there are much less coinfections with other helminths in semi-arctic regions of Western Siberia. However, it is also possible that the antigenic composition of the *O*. *felineus* is less capable of inducing TH2 responses. Some evidence for this comes from in vitro studies in which human dendritic cells cultured with *O*. *felineus* antigens lead to less TH2 skewing compared to when dendritic cells were cultured with *Schistosoma mansoni* antigens [36].

The skewing of immune responses towards TH1, TH2, and regulatory T cells can be important for understanding immunopathological processes as well as the development of cancer associated with an infection. A number of studies of humans chronically infected with, for example, schistosomes or geohelminths have shown the induction of regulatory T [37,38] and B cells [39] by these parasites. These immune regulatory cells can be involved in the suppression of responses to unrelated antigens [40,41]. A study conducted in the Tomsk region has shown an inverse association between *O*. *felineus* and responses to allergens [11], supporting the notion that it would be worthwhile to investigate whether Opisthorchiidae are able to induce regulatory cells. This is of particular importance also because of the possible link to the development of cancer. Prognosis of cancer is poor if regulatory T cells are found in tumours and a number of studies in experimental models have indicated that regulatory T cells are associated with faster tumour growth [42,43]. Whether *O*. *viverrini* and *C*. *sinensis* bear molecules that are able to induce regulatory T or B cells should be investigated. Recently, the excretory/secretory (E/S) antigens of a nematode, *Heligmosomoides polygyrus*, have been shown to drive strong regulatory T cell responses. Neutralisation of these E/S antigens resulted in complete elimination of the worms [44]. This exciting approach could also be considered for driving immunity to Opisthorchiidae as well as fighting against the associated bile duct cancer.

A presentation by Thirumalaisamy Velavan, representing the Institute of Tropical Medicine, University of Tuebingen, Germany, indicated the importance of studies regarding the innate immune components of the complement system and its interaction with Opisthorchiidae. *O*. *felineus* has an outer syncytial cytoplasmic layer as teguments, such as in other trematodes. Earlier studies have demonstrated that these trematode teguments are made up of D-mannose/D-glucose, N-acetyl-D-glucosamine/sialic acid, D-galactose, and N-acetyl-D-galactosamine residues on the glycocalyx of the adult tegument and are expressed at all developmental stages. These glycoconjugates serve as pathogen-associated molecular patterns (PAMPs) for immune recognition and subsequent complement-mediated killing [45–47]. Two innate immune recognition elements of the complement system, the mannose-binding lectin and ficolins, were earlier shown to influence the infection outcome in *S*. *haematobium* that causes urinary bladder cancer [48,49]. Additionally, the functional variants of these *MBL2* and *FCN2* genes were established to modulate the circulating serum levels and the binding capacity to the parasite surface, thus leading to impaired recognition [50]. Hence, investigation of the lectin pathway proteins during *Opisthorchis* infection might help in better understanding the interactions between the host and the parasite during an establishment of active infection.

Indeed, the question of genetic regulation of susceptibility to Opisthorchiidae and genetic control of the development of associated pathology requires attention. Maxim Freidin from the Research Institute for Medical Genetics, Russia, and Royal Brompton Hospital, United Kingdom, showed the results of a pilot gene–environment interaction study in the Tomsk population that identified *O*. *felineus* infection as an important modifier of associations between TH1/TH2-regulating genes and allergic disease. In particular, it was shown that *O*. *felineus* infection diminishes the risk of atopic bronchial asthma associated with the polymorphisms of the *SOCS5* and *IFNG* genes [4]. The allele specific gene expression was found to be modified by the presence of *O*. *felineus* antigens, thus providing a functional clue for the mechanisms of the identified gene–environment interaction. These studies form a paradigm for assessing whether tissue pathology/cancer development is controlled by inflammation as a result of gene–environment interaction.

## Diagnosis

Attempts to use molecular approaches to detect *O*. *felineus* in hospital-based studies in Europe where infections are found sporadically have been described [51]. A presentation by Vasily Petrenko from Medical Biological Union and Institute of Cytology and Genetics, Novosibirsk, Russia, showed promising results on the detection of four Opisthorchiidae flukes, *O*. *felineus* in particular, by Taqman-based real-time PCR with a high degree of sensitivity and specificity when working with faeces from hamsters infected with local strains. These results call for large-scale validation studies similar to ongoing activities regarding the molecular diagnosis of *O*. *viverrini* and *C*. *sinensis* and reliable discrimination of *O*. *felineus* infection [2]. Field applicability of these tests was discussed in terms of providing point of care diagnostic assays. Thirumalaisamy Velavan presented a new molecular diagnostic approach for rapid detection of parasites using the Loop-mediated isothermal amplification (LAMP) method [52]. These methodologies were established for *O*. *viverrini* targeting the internal transcribed spacer 1 (ITS1) in ribosomal DNA for specific amplification [53]. LAMP methodologies are simple, sensitive, specific, and faster than PCR, requiring minimal processing and instrumentation, with results available by reading with the naked eye. The development of such a LAMP methodology for *O*. *felineus* is being established.

## Parasite Biology and Drug Targets

The research group of the Institute of Cytology and Genetics in Novosibirsk, under the leadership of Aleksei Katokhin, has conducted phylogeographic genetics studies of Opisthorchiidae flukes in order to compare their genetic diversities and species histories [54]. These studies are aimed at explaining pronounced differences in capacities of the three flukes to parasitize in various mammals and shedding light on the degree of zoonosis of the different species [2]. These studies might help integrated control programs and further our fundamental understanding of biological processes.

Mariya Pakharukova and Viatcheslav Mordvinov, also at the Institute of Cytology and Genetics, Novosibirsk, presented data on the structure and functional organization of xenobiotic biotransformation system in *O*. *felineus* [55]. The system participates, probably, in *O*. *felineus* metabolism and in the transport of endogenous and exogenous substrates. Importantly, cytochrome P450 is expressed differentially through the life cycle of *O*. *felineus* and is present in *O*. *felineus* in higher amounts than in *O*. *viverrini*. The question is whether this could have any relationship to synthesis of proinflammatory and potentially carcinogenic compounds, e.g., oxysterol-like, catechol estrogen quinone-like, etc., released by the flukes [56,57]. Novel data were also presented on studies on praziquantel effects in the *O*. *felineus* hamster model and in vitro on juvenile and adult worms, particularly, about parasite motility, viability, and tegument damage after praziquantel treatment.

## Concluding Remarks: Call for Expansion of the Consortium

The data presented and discussed led us to the conclusion that there are many groups with overlapping interests and relevant expertise that are willing to work together towards the common aim of controlling liver fluke infections and associated pathologies worldwide. Most importantly, it was stressed that this is a starting point for inviting other groups actively working in the field of liver fluke research to join the Tomsk Opisthorchiasis Consortium by contacting Ludmila Ogorodova from Siberian State Medical University, Tomsk, Russia (topic.consortium@gmail.com). In particular, since there was only limited discussion of the current state of research in the area of clonorchiasis, specialists from this field would also be especially welcome.

The Tropical Disease Research Laboratory at Khon Kaen University has a successful control program, the so called “Lawa model,” based on using the EcoHealth approach at the lake Lawa region of Northeast Thailand [58]. It was agreed that this control model will be taken as a template and modified according to local health care systems and cultural sensitivities in other endemic areas, but not differing in essence of involving trans-disciplinary and stakeholder participation at every level.

One of the first issues to be addressed is to understand the knowledge gap on the prevalence of *O*. *felineus* infection in Russia and the extent of the related morbidity; in particular, whether an *O*. *felineus* infection is associated with an increased risk of bile duct cancer. In parallel, activities that facilitate faster development of validated diagnostic tests to accurately detect *O*. *felineus* infection will be stimulated and treatment efficacies of currently used drugs verified, while groups will work together in order to strengthen the scientific and technological skills needed to understand the host–parasite interaction in terms of immune-pathogenesis and regulation. Finally, the overarching, fundamental molecular biological efforts to identify new drug targets or new preventive and therapeutic measures will be stimulated by combining expertise and sharing biological material available within the Consortium.

**Fig 1:**
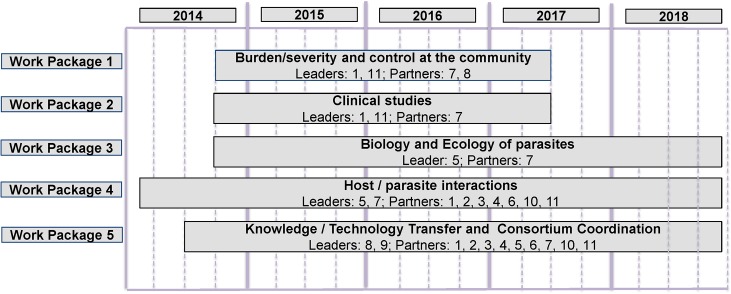
Consortium high level timelines/activities. 1. Siberian State Medical University, Tomsk, Russian Federation, 2. Center for Proteomics and Metabolomics, Leiden University Medical Center, Leiden, the Netherlands, 3. Department of Parasitology and Leiden Parasite Immunology Group, Leiden University Medical Center, Leiden, the Netherlands, 4. George Washington University Medical Center, United States, 5. Institute of Cytology and Genetics, Siberian Branch of the Russian Academy of Sciences, Novosibirsk, Russian Federation, 6. Institute of Tropical Medicine, University of Tübingen, Germany, 7. Khon Kaen University, Khon Kaen, Thailand, 8. Pfizer LLC, Moscow, Russian Federation, 9. ReMedys Foundation, Geneva, Switzerland, 10. Royal Brompton Hospital, United Kingdom; Research Institute for Medical Genetics, Tomsk, Russian Federation, 11. Swiss Tropical and Public Health Institute, Basel, Switzerland.

The Consortium shall operate through, and expand towards, several parallel research collaborative activities/work packages, depicted in the high level roadmap (Fig. 1), such as: burden/severity and control at the community; clinical studies; biology, and ecology of parasites; host–parasite interactions; knowledge/technology transfer.

These activities are detailed in an under-development project plan. The success of the Consortium shall be measured upon the delivery of research results, and, in long-term, through the applicability of these results to the society. Thus, care shall be given to the technology transfer, through all available and feasible mechanisms, bridging the gap between basic research and industrialization. To secure the planning, the establishment, the coordination, the advancement, the milestones reaching, and, finally, the knowledge/technology transfer of the results, the on-board availability of expertise in program management and industrialization will be prioritized. This can be through entities with industrial expertise such as Pfizer and the ReMedys Foundation, who are both already among the founding members of the Consortium. (Pfizer strives to positively impact the health of people around the world. Pfizer’s corporate social investment strategy focuses on leveraging the full range of the company's resources—people, skills, expertise, and funding—to broaden access to medicines and strengthen health care delivery for underserved people around the world [http://www.pfizer.com/responsibility/global_health/global_health]. ReMedys is a not-for-profit entity, founded by biopharmaceutical industry experts, intending to implement a novel, highly collaborative approach bridging the gap of translational Research and Development [R&D]. ReMedys acts as the translational R&D arm and hub for top research institutions, patient groups, and clinicians. Via building and coordinating customized patient centric alliances, ReMedys brings together all resources needed to advance, and make available to patients with high unmet need, promising therapeutic projects [http://remedys.net].)

The founding members of the Consortium have signed a Memorandum of Understanding, which shall be available for the signature of research groups interested to become new members.

The overall program shall be regulated by a Consortium agreement that is under establishment, to which new members shall be invited to join and participate. The funding for the Consortium shall be secured through national and international grants-associated activities by the members. As described, the Consortium aims to engage into a strategic collaborative study on a serious, but highly neglected, infectious disease, which is responsible for a heavy socioeconomic burden including cancer. The study shall concentrate, as well, into sensitive populations such as children, and, hence, expects to deliver findings with a high socioeconomic impact. Thus, the Consortium, with appropriate tools, can develop enhanced public awareness through the patient, the doctor, the researcher, the grant sponsor, and the government. Such an awareness shall support its funding through, as well, public, philanthropic, international NGOs; corporate responsibility partnerships; and so forth. Other funding mechanisms, such as crowd funding, in which all the Consortium members can participate, shall be explored.
